# Association between health-related physical fitness indicators and working ability: a systematic review

**DOI:** 10.1093/joccuh/uiad006

**Published:** 2023-11-08

**Authors:** Veruscka Leso, Luca Scalfi, Angela Giordano, Liberata Reppuccia, Davide Guarino, Mauro Fedele, Ivo Iavicoli

**Affiliations:** Department of Public Health, Section of Occupational Medicine, University of Naples Federico II, Via S. Pansini 5, 80131 Naples, Italy; Department of Public Health, Federico II University Hospital, Via S. Pansini 5, 80131 Naples, Italy; Department of Public Health, Section of Occupational Medicine, University of Naples Federico II, Via S. Pansini 5, 80131 Naples, Italy; Department of Public Health, Section of Occupational Medicine, University of Naples Federico II, Via S. Pansini 5, 80131 Naples, Italy; Department of Public Health, Section of Occupational Medicine, University of Naples Federico II, Via S. Pansini 5, 80131 Naples, Italy; Department of Public Health, Section of Occupational Medicine, University of Naples Federico II, Via S. Pansini 5, 80131 Naples, Italy; Department of Public Health, Section of Occupational Medicine, University of Naples Federico II, Via S. Pansini 5, 80131 Naples, Italy

**Keywords:** physical performance, work capacity, muscle strength, work productivity, risk assessment and management, health promotion

## Abstract

**Objectives:** Work ability (WA) reflects a balance between work demands and an individual’s ability to meet them. It is influenced by several occupational and health-related factors including the individual’s physical fitness (PF). Therefore, the aim of the present study was to provide an overview of the possible relationship between PF measures and the individual’s WA.

**Methods**: A systematic review of studies published up to December 1, 2022 and available in PubMed, Scopus, and ISI Web of Science databases, was performed. Results have been summarized according to the specific PF parameter explored.

**Results:** The 14 reviewed studies, enrolling 47 to 1005 workers, all showed a satisfactory methodological quality. Some positive evidence emerged for a possible association between changes in aerobic capacity, walking speed, balance, flexibility, muscle strength, and WA perception. However, the limited number of studies, their cross-sectional design, the different PF performance indicators, populations, and job tasks explored prevented definite conclusions.

**Conclusions:** Future longitudinal studies should be planned to confirm such positive results and identify PF indicators better predictive for changes in the WA of employees engaged in specific job tasks, particularly in physically demanding activities. This may be helpful to include PF performance tests in occupational health practice as an integrated part of risk assessment and management strategies as well as in health and well-being promotion plans.

## Teaser

**Key points**

- Work ability can be affected by several occupational and health-related factors including the individual’s physical fitness.
- The present review provides some evidence for a possible association between changes in aerobic capacity, walking speed, balance, flexibility, muscle strength, and work ability perception.
- The association between physical fitness and work ability is crucial for defining susceptible individuals, tailoring specific interventions to improve their physical fitness, and preparing them to face their work-related tasks and occupational risks.
- To include physical fitness performance testing in occupational health practice may be part of effective occupational health promotion strategies and can be important to inform suitable strategies for occupational risk assessment and management, to promote health and well-being, and to favor active aging of the workforce.

## 1. Introduction

The American College of Sports Medicine (ACSM) defined “physical fitness” (PF) as the set of qualities and properties that people have or develop in relation to the ability to perform physical and daily life activities.^1–3^

From a different perspective, work ability (WA) is a construct that reflects the balance between work demands and an individual’s ability to meet them.^4^ In addition to the association with nearly all factors of life including health, workplace environment, and the extra-occupational dimensions, it seems evident that PF can affect WA.^5^ A poor WA is predictive of sick leave, early retirement, disability pension,^6–8^ work-related stress, depression, and emotional exhaustion.^9–12^ Conversely, a good WA is associated with high quality of work, high productivity, and enjoyment of time on job, thus predicting a good quality of life and well-being as well as an active and meaningful retirement.^13^

Whereas the effect of age, gender, education, employment status, and occupation as well as socioeconomic status have been explored,^4^ only few studies have investigated the impact of PF on WA. This seems even more important considering the aging of the workforce, a major concern for public health policies and responsible for health worsening, in terms of physiological and cognitive abilities, that may decrease WA.^14^

If a worker’s physical capacity is unable to meet the demands of the job, due to a progressive deterioration of various components of PF, he/she can suffer from excessive fatigue and worse WA perception, thus leading to poor productivity and increased risk of workplace accidents.^15^^,^^16^

Against this background, the aim of this review was to comprehensively assess the state of knowledge concerning the possible relationship between components of PF, including aerobic and muscular fitness, joint flexibility, and balance tasks, and WA. Given an holistic approach to the health of workers, this overview may provide data that are useful to include PF assessment in occupational health practice as part of health and well-being promotion plans. This may help workers to remain in the workforce and improve their productivity, supporting a long-term sustainable WA.

## 2. Materials and methods

A systematic review was performed according to the Preferred Reporting Items for Systematic Reviews and Meta-Analyses Statement (PRISMA) criteria, although it was not registered in international databases (Supplementary material 1-3).^17^ The research was carried out in PubMed, Scopus, and ISI Web of Science databases to identify studies, published up to December 1, 2022 and exploring the relationship between PF measures and WA. In all 3 databases, the full line search strategy included the term (“Physical fitness”) combined, through the Boolean operator “AND”, with the term (“work ability”). All titles and abstracts were evaluated by 2 of the authors, who performed a selection of articles relevant to the review. Inclusion criteria regarded both cross-sectional and prospective cohort studies, published in English, addressing the influence of different PF parameters on the WA. The full texts of the eligible articles have been screened for inclusion by 2 researchers independently. In case of disagreement, in this phase, consensus on inclusion and exclusion was reached by discussion and, if necessary, a third researcher was consulted. The citation pool of relevant publications identified in the literature search was further enlarged by searching online for more specific terms related to the PF evaluation, for example, “aerobic capacity,” “walking speed,” “balance,” “flexibility,” “muscle strength,” and “hand grip strength” (HGS) combined with “work ability” and assessing the reference list accompanying the selected articles.

### 2.1. Physical fitness

Physical fitness has been defined as “the ability to carry out daily tasks with vigor and alertness, without undue fatigue and with ample energy to enjoy leisure-time pursuits and to meet unforeseen emergencies.”^18^ A number of measurable components contribute to PF. These include balance, agility, coordination, speed, power, and reactivity, as well as those components more strictly associated with health and well-being (health-related fitness), namely, cardio-respiratory endurance, muscular endurance and strength, body composition, and flexibility.^2^ Therefore, we attempted to define the possible impact that changes in measurable PF factors may have on the WA of subjects employed in different occupational settings.

### 2.2. Work ability

In occupational health, the WA concept refers to the balance between a person’s resources, such as health and functional abilities, education, and competence, as well as values and attitudes and work demands.^19^ These latter embrace the work environment and community, as well as the actual content, demands, and organization of work. Considering that the WA evaluation is a challenging issue and that many measures have been proposed, we have chosen to focus on studies using the Work Ability Index (WAI)^12^^,^^19^ or the Work Ability Score (WAS)^20^^,^^21^ questionnaires, known for their well-established predictive validity in WA assessment. The WAI^19^^,^^22^ is a summary measure of 7 items including: individuals’ current ability to work in comparison with their best years of life; their ability to work concerning their demand for work; the number of diagnosed diseases or limitations from which they suffer; their estimated impairments due to diseases/abilities or limitations; the number of sick leaves they have taken during the previous year; self-prognosis of WA for the next 2 years; and mental resources. The WAI score ranges from 7 to 49 points. Scores of 7-27, 28-36, 37-43, and 44-49 correspond to low, moderate, good, and excellent WA, respectively.^23^^,^^24^ To avoid workers’ difficulties in completing all the numerous items of the WAI, the WAS, the first item of the WAI, was proposed and adopted as a valid, simple, and suitable measurement for WA.^24^ It scores from 0 (“completely unable to work”) to 10 (“work ability at its best”). The obtained results using the WAI and WAS are similar and they have satisfactory convergent validity.^21^

### 2.3. Data analysis and reporting

Key information about the included studies was collected in a standardized data extraction form independently by 3 of the authors, and extracted data were then compared to exclude any possible inaccuracy during the process. They independently evaluated also the quality of the selected studies using the Newcastle-Ottawa Quality Assessment Scale for case control and cohort studies or adapted for cross-sectional ones.^25^^,^^26^ Based on a maximum of 9 points attributable within 3 different sections, such as selection, comparability, and outcome, a range scale was adopted, going from satisfactory studies with 5-6 points, good studies for 7-8 points, and very good studies for 9-10 points. Scores less than 4 categorized studies as unsatisfactory. When there was disagreement on the evaluation, the remaining authors also reviewed the article, and the judgment made by most of the reviewers determined the quality rating. In the “Results” section, the findings of the studies have been summarized with the aim of defining the impact of PF on WA according to the correlation or odds ratio results reported in the included studies. In Table 1, the population of the reviewed studies is defined by reporting the number of workers considered in individual studies, age, sex, and type of work performed, including administrative and managerial work, “white-collar workers,” as well as “blue-collar” ones, including workers engaged in significant physical effort at work. The anthropometric measurements and tests used to assess the PF performance and the scores obtained are reported. Finally, the results of the individual studies are also summarized to provide readers with a suitable overview of the relationship between the investigated PF parameters and the perceived WA.

**Table 1:** Studies assessing the relationship between physical fitness status and work ability.

**Study location (analyzed period)**	**Study design**	**Population investigated (number and age)**	**Type of work**	**Physical fitness and work ability assessment**	**Results**	**Quality rating** ^**a**^	**Reference**
Finland	Cross-sectional	Workers: 137; 65 F, 72 M; age: 46–62 years Individuals affected by cardiovascular (*n* = 23) and musculoskeletal diseases (*n* = 44)	Municipal employees	*Physical fitness:* ✓Cardiorespiratory tests: ✓Bicycle ergometer test ✓Maximal oxygen consumption ✓Maximal workload ✓Musculoskeletal tests: ✓HGS ✓Trunk flexion and extension strength; muscular endurance (sit-up test) ✓Forward and sideward back bending ✓Leg mobility *Work ability:* WAI	Maximal workload (*r* = 0.18), sideward back bending (*r* = 0.17), and leg mobility (*r* = −0.16) were not significantly correlated with the WAI HGS (*r* = 0.27; *P* < .01); trunk flexion strength (*r* = 0.32; *P* < .01); trunk extensions strength (*r* = 0.30; *P* < .01); trunk muscular endurance (*r* = 0.37; *P* < .001) were positively correlated with the WAI Sideward back bending (*r* = 0.51; *P* < .05) and trunk muscular endurance (*r* = 0.38; *P*< .01) were correlated with the WAI in cardiovascular and musculoskeletal disease-affected workers, respectively	Satisfactory	Nygård et al^38^
Helsinki, Finland (1993-1998)	Prospective cohort	Workers: 132 F (mean age: 41.0 years) Age subgroups: 21-35 (*n* = 42); 36-44 (*n* = 34); 45-59 (*n* = 56)	Home care workers caring for elderly people	*Physical fitness:* ✓BMI ✓Sit-up; squatting; weight-lifting; HGS ✓Trunk and knee extensions ✓Sit-and-reach test ✓Trunk side-bending ✓Balance test ✓Aerobic capacity: V̇o_2max_ (L/min) *Work ability:* WAI	BMI was a significant predictor of poor WA: BMI >30 (OR 7.51; 95% CI, 1.88-30.0) Risk for a decreasing WA over a 5-year period of follow-up: ✓Trunk side-bending: average (OR 4.56; 95% CI, 1.18-17.86); poor (OR 4.07; 95% CI, 1.15-14.38) ✓Balance test: poor (OR 6.53; 95% CI, 1.84-23.25) ✓Squatting: poor (OR 3.98; 95% CI, 1.11-14.23) ✓Sit-up: average (OR 3.66; 95% CI, 1.30-10.30), poor (OR 8.88; 95% CI, 2.42-32.60) ✓Weight-lifting: poor (OR 4.63; 95% CI, 1.49-14.34) ✓Knee extension strength: poor (OR 4.24; 95% CI, 1.19-15.10) ✓V̇o_2max_: average (OR 3.07; 95% CI, 1.05-8.99)	Satisfactory	Pohjonen^28^

**Table 1:** Continued.

**Study location (analyzed period)**	**Study design**	**Population investigated (number and age)**	**Type of work**	**Physical fitness and work ability assessment**	**Results**	**Quality rating** ^**a**^	**Reference**
Lódź, Poland	Cross-sectional	Workers: 271; mean age: 42.9 years. Professionally active: 198 (103 M, 95 F)	Physical job: 49; mental job: 105; mixed job: 44	*Physical fitness:* ✓Treadmill ✓Total weekly energy expenditure on leisure-time physical activity ✓Total weekly time expenditure on leisure-time physical activity ✓BMI ✓V̇o_2max_ (cardiorespiratory fitness evaluation) *Work ability:* WAI	Leisure-time physical activity was positively correlated with WAI (*r* = 0.3, *P* < .0001); V̇o_2max_ was positively correlated with WAI (*r* = 0.4, *P* < .0001) Age and BMI were negatively correlated with WAI (*r* = −0.6, *P* < .0001; *r* = −0.3, *P*< .0001, respectively)	Very good	Kaleta et al^31^
Kuopio, Finland 1996-1999	Prospective cohort	Workers: 135 M; age: 33–56 years	Firefighters	*Physical fitness:* ✓Functional balance ✓Sway amplitude ✓Sway velocity ✓Perceived balance *Work ability:* WAI, PWA	Poor-moderate perceived balance: positively correlated to the WAI and PWA (analysis performed in 1996; OR 9.8; 95% CI, 3.8-24.9; OR 5.5; 95% CI, 2.2-13.7, respectively), and to WAI and PWA (analysis performed in 1999; OR 4.9; 95% CI, 2-12.3 and OR 5.5; 95% CI, 2.2-13.9, respectively) Poor functional balance and average sway amplitude: significantly associated with WAI (analysis performed in 1999; OR 3.6; 95% CI, 1-12.7 and OR 2.7; 95% CI, 1-7.2, respectively)	Good	Punakallio et al^39^
Finland	Cross-sectional	Workers: 104 M; age: 45–55 years	Employees in construction and manufacturing industries	*Physical fitness:* ✓V̇o_2max_ ✓Maximal load ✓2-km walking time ✓V̇o_2max_ by 2-km walking test ✓2-km fitness index *Work ability:* WAI (assessed in a subgroup of 51 subjects)	V̇o_2max_ by 2-km walking test (*r* = 0.033; *P* < .05), and 2-km fitness index (*r* = 0.030; *P* < .05) were positively correlated with the WAI	Satisfactory	Sörensen et al^27^
	Cross-sectional			*Physical fitness:* ✓HGS dynamic lifting with both arms ✓Back endurance ✓Sit-up test ✓Squatting test *Work ability:* WAI (assessed in a subgroup of 43 subjects)	Dynamic lifting test with both arms: weak, but significant positive correlation with the WAI (right arm: *r* = 0.31; left arm: *r* = 0.34; *P* < .05 for both the associations) No significant correlations with the other tests	Satisfactory	Smolander et al^29^

**Table 1:** Continued.

**Study location (analyzed period)**	**Study design**	**Population investigated (number and age)**	**Type of work**	**Physical fitness and work ability assessment**	**Results**	**Quality rating** ^**a**^	**Reference**
Gothenburg, Sweden 2007-2012	Cross-sectional	Workers: 47 (11 F, 36 M); age: 24-69 years Women with HAVS: 11	Manual workers exposed to HAV	*Physical fitness:* ✓HGS ✓Key grip/pinch key ✓Pinch 3-chuck ✓Purdue pegboard *Work ability:* WAI	The HGS (*r* = 0.32; 95% CI, 0.028-0.56; *P* = .033); the key grip/ pinch key (*r* = 0.33; 95% CI, 0.049-0.57; *P* = .023); the pinch 3-chuck (*r* = 0.27; 95% CI, −0.017 – 0.52; *P* = .064); and the Purdue pegboard (*r* = 0.32; 95% CI, 0.029-0.56; *P* = .032) were positively correlated with the WAI	Good	Edlund et al^33^
São Paulo, Brazil	Cross-sectional	Workers: 79 (50 F, 29 M) Age subgroups: <50 years: (*n* = 37); >50 years: (*n* = 42)	Employees of a higher education institution and of a metallurgical industry	*Physical fitness:* ✓HGS ✓Usual gait speed ✓Maximum gait speed ✓Right unipedal stance time ✓Left unipedal stance time ✓Sit-to-stand test ✓Five-step test *Work ability:* WAI	HGS was negatively correlated with the WAI in <50 year-old workers (*r* = −0.368; *P* = .027) The 5-step test was negatively correlated with the WAI in **>**50 year-old workers (*r* = −0.304; *P* < .050) The sit-to-stand test was positively correlated with the WAI in women >50 years old (*r* = 0.573; *P* < .050)	Satisfactory	Padula et al^34^
Sweden, 1997-2008	Prospective cohort	Workers: 157 M Median age: 40 years (1997); 51 years (2008)	Engineering plant employees: manual workers (*n* = 112); office workers (*n* = 45)	*Physical fitness:* ✓HGS *Work ability:* WAS, PWA, MWA	Cross-sectional results in 2008: ✓HGS L and R: positively correlated to the WAS (*r* = 0.17 and *r* = 0.17, *P* < .05, respectively) and to the PWA (*r* = 0.17 and *r* = 0.22, *P* < .05, respectively) in the total population ✓HGS R: positively correlated to the PWA (*r* = 0.22, *P* < .05) in manual workers’ group Prospective results (1997-2008): ✓HGS L and R: positively correlated to the WAS (*r* = 0.21 and *r* = 0.21; *P* < .05, respectively) and to the PWA (*r* = 0.25 and *r* = 0.30, *P* < .05, respectively) in the total population ✓HGS R: positively correlated to the PWA (*r* = 0.33, *P* < .01) in manual workers’ group; HGS L: protective effect to poor WAS (RR 0.91; 95% CI, 0.83-1.00; *P* = .04) in office workers’ group	Good	Boschman et al^35^

**Table 1:** Continued.

**Study location (analyzed period)**	**Study design**	**Population investigated (number and age)**	**Type of work**	**Physical fitness and work ability assessment**	**Results**	**Quality rating** ^**a**^	**Reference**
Vienna, Austria, 2015-2016	Cross-sectional	Seropositive RA workers: 100 (66 F) Mean age: 53 years Gainfully employed: 59	Physical job: 10 Mental job: 21 Mixed job: 28	*Physical fitness:* ✓HGS ✓Knee extensor strength (KES) ✓Sit-to-stand, balance test, walking test (SPPB) *Work ability:* WAS	The HGS (*R*^2^ = 0.31, β: 0.49, *P* < .001), the KES (*R*^2^ = 0.33, β: 0.52, *P* < .001), and the SPPB (*R*^2^ = 0.39, β: 0.5, *P* < .001) were positively corrrelated with the WAI	Good	Berner et al^32^
Virginia, USA	Cross-sectional	Workers: 312 (275 F, 37 M) Age: >18 years	Inpatient nursing staff	*Physical fitness:* ✓Walking test (10 m) *Work ability:* WAS	Maximum gait speed condition was positively associated with WAS (*r* = 0.217; *P* < .0001). Workers with a maximum gait speed of 1.83 m/s were more likely to have a moderate to poor WAS rating (*P* = .015); those with a speed of 1.83-2.04 m/s had no significant correlation with WAS; when the speed was >2.04 m/s a significant association was determined with a good-excellent WAS rating (*P* = .014) The preferred gait speed was not significantly associated with WAS (*r* = 0.109; *P* = .041).	Good	Aldridge et al^36^
Sydney, Australia (January 2014 to September 2015)	Cross-sectional	Workers: 720 (49% F, 51% M); age: 18-101 years Age subgroups: 18-44 (*n* = 240); 45-64 (*n* = 232); >65 (*n* = 248)	Blue-collar (*n* = 95); white-collar (*n* = 311); unemployed (*n* = 314)	*Physical fitness:* ✓Anthropometric measurements: BMI ✓Isometric muscle strength tests ✓Joint flexibility tests ✓Gross and fine motor function tests**:** timed up-and-down stairs test (TUDS) ✓Balance tests *Work ability:* WAS	BMI: significant negative correlation with WAS (*r* = −0.192; *P* < .001) Age: significant negative correlation with WAS (young [*r* = −0.161; *P* < .05]; middle-aged [*r* = −0.247; *P* < .01]; older [*r* = −0.341; *P* < .01]) TUDS: significant negative correlation with WAS (*r* = −0.498; *P* < .01)	Good	Lebde et al^4^
Temuco, Chile 2017	Cross-sectional	Workers: 360 Age subgroups: 40-49 years (*n* = 120); 50-59 years (*n* = 120); >60 years (*n* = 120)	Employees in public institution (university and high-complexity regional hospital)	*Physical fitness:* ✓30-second sit-to-stand test ✓1RM-leg extension ✓1RM-handgrip ✓Modified sit-and-reach test ✓Back scratch test ✓Single leg stance test ✓Timed up-and-go test *Work ability:* WAI	The 30-second sit-to-stand test (*r* = 0.13; 95% CI, 0.02-0.23, *P* = .018); the modified sit-and-reach test (*r* = 0.13, 95% CI, 0.04-0.23, *P* = .012), and the single leg stance test (*r* = 0.13, 95% CI, 0.03-0.23, *P* = .012) were positively correlated with the WAI	Very good	Marzuca-Nassr et al^37^

**Table 1:** Continued.

**Study location (analyzed period)**	**Study design**	**Population investigated (number and age)**	**Type of work**	**Physical fitness and work ability assessment**	**Results**	**Quality rating** ^**a**^	**Reference**
Spain	Cross-sectional	Workers: 1005; 712 F (mean age: 34.1 years); 293 M (mean age 35.3 years)	Physical therapists	*Physical fitness* (performed on a subgroup of 68 participants): ✓HGS ✓Biering-Sørensen back-extensor endurance test ✓Push-ups *Work ability:* WAI (assessed in a subgroup of 68 subjects)	Biering-Sørensen back-extensor endurance test: high-low performance was positively correlated to the WAI (least square mean difference:3.5; 95% CI, 0.2–6.8)HGS or number of push-ups: no significant correlations with the WAI	Good	Ezzatvar et al^30^

a The quality rating of the selected studies was addressed using the Newcastle-Ottawa Quality Assessment Scale for case control and cohort studies or adapted for cross-sectional ones.

Abbreviations: BMI, body mass index; F, female; HAV, hand-arm vibrations; HAVS, hand-arm vibrations syndrome; HGS, hand grip strength (test); KES, knee extensor strength; L, left; M, male; MWA, perceived mental work ability; OR, odds ratio; PWA, perceived physical work ability; R, right; RA, rheumatoid arthritis; RM, repetition maximum; RR, relative risk; SPPB, short physical performance battery score; TUDS, timed up-and-down stairs (test); V̇o_2max_, maximal oxygen consumption (mL/kg/min); WA, work ability; WAI, Work Ability Index; WAS, Work Ability Score.

## 3. Results

**Figure 1:**
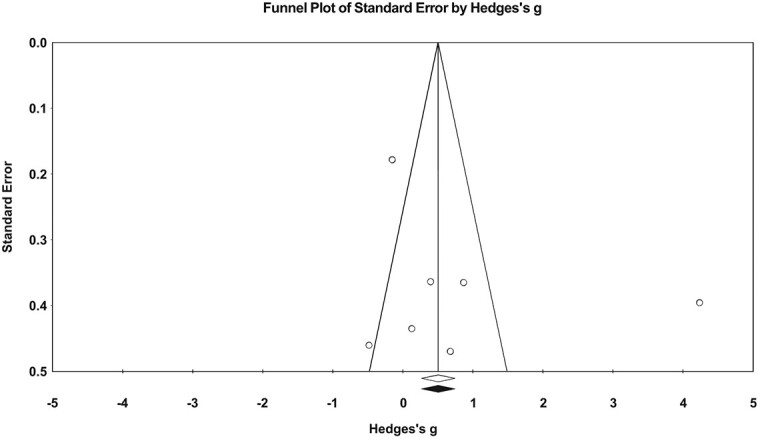
Flow diagram of literature search.

The first step of the search strategy retrieved 59, 63, and 37 records on PubMed, Scopus, and ISI Web of Science databases, respectively (Figure 1). After removal of duplicates, 2 researchers independently reviewed titles and abstracts of all identified articles (79) and discussed inconsistencies until a consensus was obtained. A total of 72 articles were excluded, 50 studies were not included because they were off topic for title and abstract analysis, 13 because they were review articles, letters to the Editor, conference abstracts, and book chapters, and 9 because they were published in languages other than English (Supplementary material 1). Then, the full texts of the remaining 7 articles were screened for inclusion^4^^,^^27–32^ (Supplementary material 1). The additional search allowed the inclusion of 7 additional eligible papers, 5 from the enlarged search online^33–37^ and 2 from the analysis of the reference lists.^38^^,^^39^ Overall, our search retrieved a total of 14 articles suitable for review.

The articles, published between 1991 and 2022, analyzed the relationships between PF and WA. All the studies showed at least a satisfactory methodological quality, in terms of design, conduct, and analysis, with 7 out of 14 classified as good (*n* = 5) or very good (*n* = 2), thus preventing risk of biases in findings. Most investigations were conducted in Scandinavia, particularly in Finland,^27–29^^,^^31^^,^^38^^,^^39^ where PF assessment is part of routine practice of workplace health and well-being promotion. The reviewed studies mostly involved cohorts of healthy workers; only 1 investigation specifically engaged manual workers who claimed signs and symptoms of hand-arm vibrations syndrome (HAVS).^27^ Two studies considered the impact on WA of cardiovascular and musculoskeletal disorders^38^ or musculoskeletal pain of the upper limb.^35^ In general, the nature of study design was cross-sectional, with only 3 investigations evaluating the predictive power of PF on future WA.^28^^,^^35^^,^^39^ Physically demanding jobs were primarily investigated, because it was assumed that a good level of PF and efficiency was necessary in that case to achieve a higher WA perception. In this view, home care workers,^28^ nurses,^36^ physiotherapists,^30^ employees in the construction and manufacturing industry,^27^^,^^29^ and in fire rescue^39^ were taken into consideration. Three studies aimed to investigate differences in the relationship between PF tests and WA results in subgroups of white- and blue-collar workers.^4^^,^^34^^,^^35^ One study considered only sedentary work,^38^ and only 1 group of researchers included unemployed subjects.^4^ Work ability was mostly assessed using the WAI, whereas 3 studies utilized the WAS.^4^^,^^35^^,^^36^ The following paragraphs will attempt to summarize the results according to the relationships between different components of PF and perceived WA.

### 3.1. Aerobic capacity

Maximal oxygen uptake (V̇o_2max_), also known as functional aerobic capacity (AC), represents the maximal rate of oxygen consumption by exercising muscles and is considered the gold standard measure of the cardiorespiratory functional limit. Walking speed is the result of a complex interaction between multiple body functions including proactive and reactive postural control, lower extremity strength, AC, proprioception, and vision.^40^ Kaleta et al^31^ found a significantly positive association between V̇o_2max_ and WA in working residents of Łódź, employed as white-collars, blue-collars, and mixed activity employees. When Sörensen et al^27^ evaluated the correlation between WA and V̇o_2max_ (measured through the cycle ergometer test or estimated through the 2-km walking test) in subjects employed in construction and manufacturing industry, a significantly positive association was only demonstrated with the values estimated with the walking test method. Additionally, in female home care workers, engaged in physically demanding tasks while caring for elderly people in private or institutional homes, an unclear relation was demonstrated, as the intermediate values of V̇o_2max_ were significantly associated with an increased risk for a worse WA compared with high and low levels.^28^ Conversely, Nygård et al^38^ failed to find a significant correlation between cycle ergometer V̇o_2max_ and WAI in a group of municipal employees engaged in different physically (ie, construction workers, street cleaners, domestic helpers), mixed (ie, bus drivers, nurses), or mentally (administrative workers, technical supervisors, teachers) demanding tasks. Furthermore, the WA was correlated with the AC only in the group of healthy workers and not in those affected by musculoskeletal disorders, probably because pain affected the test, forcing the workers with musculoskeletal problems to an earlier stop.^38^

### 3.2. Walking speed

Walking speed is the result of a complex interaction between multiple body functions including proactive and reactive postural control, lower extremity strength, AC, proprioception, and vision.^34^ Walking speed, which is affected by functional capacity and overall health status, has been shown to be predictive of various outcomes, including functional dependence, frailty, cardiovascular risk, and cardiovascular as well as all-cause mortality.^41^^,^^42^ In the study by Berner et al,^32^ a group of measures including tests of gait speed, balance, and sit-to-stand were used to assess lower extremity function, and were significantly associated with WAS. In hospital nurses, Aldridge et al^36^ demonstrated that the maximum gait speed, but not the preferred gait speed, was significantly correlated with WA. Maximum speeds ≤1.83 m/s and >2.04 m/s were significantly associated with a moderate/poor and good/excellent WAS score, respectively, whereas intermediate maximum speeds (1.83-2.04 m/s) showed no significant association. Lebde et al^4^ observed significant, although weak, correlations between WAS and walking speed at 6 minutes also in the subgroup of older workers (>65 years). On the other hand, contradictory results were reported by Padula et al,^34^ who failed to find any significant association between walking speed and WAI in academic or administrative workers of a private higher education institution or employees of a metallurgical industry; in that study the small sample size could have limited the power of statistical analysis.

### 3.3. Balance indicators

Balance is the ability to maintain or recover a stable position when an external or internal factor tends to change it. It includes a static mode, defined as the condition of stability and maintenance of a position by a subject, and a dynamic one, that is, the ability to assume the most suitable posture in a movement.^43^ Pohjonen^28^ showed that poor dynamic functional balance was a strong predictor for a reduced WA over a 5-year follow-up period in female home care workers. In firefighters, Punakallio et al,^39^ over a 3-year period of investigation (1996-1999), demonstrated that poor-to-moderate perceived balance was strongly associated with decreased WAI and perceived physical work ability (PWA), and a significant negative predictor of a lower WAI score 3 years later. Consistent with these results, Lebde et al^4^ demonstrated weak but significant associations of both static and dynamic balance tests with WA also in the oldest subgroup of investigated workers (>65 years). In a cohort of university and hospital employees, Marzuca-Nassr et al^37^ found a weak association between WA and functional ability assessed using the static balance tests, but not the dynamic ones. Only the study carried out by Padula et al^34^ failed to find a significant correlation between static balance tests and WA perception.

### 3.4. Flexibility

Flexibility has been defined as the range of motion of muscle and connective tissues at the level of a specific joint or group of joints.^44^ Pohjonen^28^ found that average or poor scores of lateral trunk flexibility in home care workers were associated with a high risk of reduced WA in the next 5 years. Two studies conducted on municipal workers as well as on university and hospital employees confirmed this weak association.^37^^,^^38^ In a subgroup analysis, focusing on subjects affected by cardiovascular diseases, lateral trunk flexibility was the only parameter that correlated with WA.^38^ A more comprehensive battery of tests, including the range of motion of neck flexion/extension, shoulder internal/external rotation, elbow flexion/extension, hip flexion/internal/external rotation, knee flexion/extension, and ankle plantarflexion/dorsiflexion, used to assess workers’ joint flexibility was proposed in the study by Lebde et al^4^ of a large cohort of 720 workers divided into different age groups. All tests correlated with the WA of the overall population, except those related to the shoulder internal rotation and elbow flexion.

### 3.5. Muscle strength

Muscle strength is the ability of a skeletal muscle to produce force, which can be measured during a single maximal voluntary contraction and under a defined set of controlled conditions, which include pattern of movement, type of muscle contraction (concentric, isometric, or eccentric), and speed of contraction.^45^^,^^46^

### 3.6. Hand grip strength

Hand grip strength (HGS) showed a weak correlation with WA in municipal employees,^38^ but not in workers employed in physically active jobs in manufacturing and construction industries.^29^ In patients presenting with vascular and/or neurological symptoms from HAVS, all tests of hand strength and dexterity, and finger strength were shown to be predictive of WA.^33^ Padula et al^34^ reported that the HGS was negatively associated with the WA in individuals aged 50 years or less, whereas a nonsignificant correlation with WA was demonstrated by Marzuca-Nassr et al.^37^ In the study by Lebde et al,^4^ HGS showed a weak association with WAS in the total population and in the younger group, but not in the middle-aged and older adult individuals. In physiotherapists, Ezzatvar et al^30^ demonstrated that the HGS did not correlate with WA. Finally, Boschman et al^35^ evaluated the feasibility of using the HGS as an indicator of WA in blue-collar workers, that is, those engaged in unskilled, manual jobs not requiring specific training, and in white-collar workers, namely those involved in skilled jobs requiring a certain formal education. In the total enrolled cohort, both left and right HGS weakly correlated with WAS and PWA, and this association was confirmed 11 years later on the same prospective population (with 18% loss from baseline). In a subgroup analysis, workers with poor HGS of the right hand had a significantly increased risk of poor PWA both at the time of the survey as well as after in a follow-up of 11 years.

### 3.7. Sit-to-stand and step tests

In the study by Pohjonen,^28^ home care workers who showed medium scores at the sit-to-stand test demonstrated a 3.7-fold risk of a reduction in WA compared with those who had a high good score, whereas those with a low score showed an 8.9-fold increased risk over a 5-year follow-up. Padula et al^34^ found a significant correlation between the 5-step test with WA perception for the subgroup of workers aged >50 years employed in metallurgical industry. In contrast, the study by Smolander et al^29^ of male workers aged 45-55 years failed to find an association between the sit-to-stand test and WA. Similar results were reported by Marzuca-Nassr et al^37^ in university and hospital workers.

### 3.8. Other strength tests

Nygård et al^38^ found in municipal employees that all tests of back isometric strength correlated with WA. In particular, the trunk muscular endurance was the strongest predictor of WAI score in female participants and in the subgroup of workers with musculoskeletal diseases. A more recent study demonstrated a correlation between all the strength measures investigated, from the shoulder internal rotation strength to the knee extension strength, and WAS values.^4^ Conversely, the study of Smolander et al^29^ showed no correlation between back endurance and WA in workers employed in physically active work in manufacturing and construction industries. Padula et al,^34^ studying workers of an educational institute and a metal industry, found no significant differences in the relationship between muscle efficiency and WA in subjects aged <50 and ≥50 years. In physiotherapists, Ezzatvar et al^30^ demonstrated a positive association between the high/low performance on the Biering-Sörensen test, aimed to assess the back extensor muscle strength, and WAI. Smolander et al^29^ could observe a weak, but significant, correlation between the dynamic lifting right and left arm test with WAI in male workers. Ezzatvar et al^30^ failed to demonstrate a significant correlation between the push-up test used for the dynamic physical assessment and WA in physiotherapists. For workers exposed to HAV, a correlation of the pinch key test with the WAI score was found whereas no correlation could be determined with the pinch 3-chuck test.^33^ Interestingly, concerning the predictive role of PF on WA changes, a poor performance in weight-bearing strength and knee extension exercises correlated in home caregivers with an increased risk of WA decline after 5 years.^27^

## 4. Discussion

This review is, to our knowledge, the first attempt to provide an overview on both the relationships between WA perception and workers’ PF and the individual and work-related factors potentially affecting such relationships. Overall, although positive findings have been reported, no definite association could be defined between PF parameters and WA.

This may be due to the limited number of available studies and to some critical issues to be considered, such as the variability between study groups in terms of sex, age, or homogeneity. Actually, only a few studies included groups of different age^4^^,^^34^ or workers affected by cardiovascular or musculoskeletal diseases,^27^^,^^38^ thus making it impossible to analyze the impact of aging or chronic pathologies on PF and WA perception.

As for occupational features, no clear difference emerged when physically demanding jobs were compared with the sedentary ones in terms of AC and PF.^27^^,^^28^^,^^31^^,^^33^^,^^34^^,^^38^ Of note, all the retrieved studies failed to provide a clear classification of job activities according to an objective measure of “workload”; only a qualitative description of the job tasks, that is, sedentary or highly physically demanding ones, was included. This limitation should be overcome in the future to better assess the influence of the workload itself on PF and WA. This may imply a deeper assessment of physical workload risk factors in the workplace.^44^ This should include the definition of the type of physical efforts applied: manual material handling, working in awkward postures, repetitive work, work involving high exertion and/or exposure to force, as well as the employment of questionnaires and workers’ self reported data on physical workload. Additionally, the use of checklists to identify specific physical workload types; observational methods to more precisely assess such risk factors (eg, the Finnish Ovako Working posture Assessment System method, the Key Indicator Methods, the the OCcupation Repetitive Action (OCRA) method/OCRA checklist); and also the performance of measurements of physical workload directly at the workplace (eg, the computer-aided recording and long-term analysis of musculoskeletal workloads measuring method [CUELA]) or in the laboratory can be useful to achieve a more objective evaluation of the risk in relation to PF and WA.^45–49^ Apart from physical workload, while performing their job tasks workers may experience a series of other risk factors, including physical (eg, total body or hand-arm vibrations), organizational (eg, shift and night work), and psychosocial risks, primarily due to work-related stress, that may all significantly impact both their PF and WA perception and reporting. Unfortunately, the reviewed studies did not provide reliable information on such risk factors, making it impossible to analyze the potential influence of occupational factors on the relationship between PF and WA.

As another point, a suitable evaluation in occupational settings should include a “composite” approach aimed to address different PF aspects. Defining those components that may be more reliable predictors of WA is even more important for workers in physically demanding jobs, and for those affected by diseases that can impact musculoskeletal capacity. The selection of suitable tests should be performed considering the characteristics of the specific job tasks and the musculoskeletal units involved. For instance, the nonsignificant predictive role of V̇o_2max_ on WA^38^ compared with the positive relationship found for the walking test at 2 km^33^ could be ascribed to the “representativeness” of the test with respect to specific job requests. Because manual tasks may often involve walking, transporting, or climbing stairs, the walking test may be more convenient and easier to predict WA.^26^ Similarly, the physical function of muscles of the lower limbs, most clearly affected by the occupational physical demand, could act as an appropriate predictor of WA.^34^ In another study,^29^ the dynamic lifting tests for the upper limbs more significantly correlated with the WAI scores compared with other muscle performance tests as the explored job tasks consisted in manual handling of materials and tools, as well as machinery operations that may have more clearly impacted the upper limbs.

Some contradictory results have emerged related to the aim of this review. In some cases, in fact, the PF parameters investigated were not significantly associated with WA, possibly because they were not related to the tasks expected for this occupation,^29^ but also because of a potential “healthy worker effect”^28^^,^^35^: in such a case a lower PF performance may be predictive for poor WA, disability, and in turn, early retirement.^35^ When more homogeneous results have been reported,^4^^,^^28^^,^^32^^,^^37^^,^^39^ it could be suitable to consider the possibility to include such PF tests into the health surveillance programs. This may be helpful both to prevent the risk of injuries, and to promote workers’ health and WA.

The relationships between PF and WA were addressed in a few longitudinal studies.^27^^,^^28^^,^^39^ Concerning the predictive role of PF parameters with respect to future WA, one study^35^ showed a relationship between musculoskeletal health and WA depending on occupation. In fact, these authors failed to find PF predictors for future poor physical WA among manual workers, whereas among office workers a lower HGS related to poor future WA. Another study,^28^ in female home care workers over a 5-year follow-up, failed to confirm HGS as a predictor of WA, despite the subjects’ physically demanding tasks. Thus, predicting WA in the far future is rather difficult based on the available evidence.

Overall, some critical issues emerged from the present review that need to be addressed in future research. Concerning the systematic review methodology, no registration in international databases was performed. This is an essential step to provide transparency in the review process and ensure that the findings of the systematic review are drawing on the best-quality evidence. However, a detailed protocol of the review was defined, as reported in the Supplementary materials, and we have included all relevant data on information sources, search strategy, selection process, and methods applied to synthesize and report results in the “Materials and methods” section to sustain the trustworthiness and applicability of review findings and help others to replicate and update results. Additionally, a proportion of included studies were retrieved through sources different from the preliminary online search, particularly revising the search strategy to include more specific terms related to the PF evaluation. This may be related to the lack of a homogeneous reference to PF, the term used in the search line, as the outcome of the investigations due to the different specificity of the tests in assessing peculiar aspects of the physical performance. This issue has been responsible for the difficulty in identifying suitable and most comprehensive search terms able to capture all the eligible studies and characterizes a limitation in the search strategy effectiveness. However, we attempted to overcome such search bias through an enlarged search that employed more specific terms related to the PF evaluation and allowed us to include additional studies and fill the gaps related to the preliminary adopted strategy. Overall, this underlines the need to more deeply explore the individual PF components to achieve a more comprehensive understanding of the impact that PF may have on WA.

The number of retrieved studies is limited. Most research done in the field of PF and WA is based on cross-sectional research and only few studies reported prospective results.^28^^,^^34^^,^^35^^,^^39^ This does not allow us to reach conclusions on PF predictors concerning changes in WA. Moreover, most of the retrieved studies come from Scandinavian countries where PF tests are included in routine occupational health practice and workplace health promotion. This provides only a preliminary and partial picture of the relationships between PF and WA. Moreover, the retrieved studies have been performed over a wide time span: this means that the working environments, methods, and tools as well as the workforce itself, have changed, and this could have been responsible for a diverse WA perception and limited the comparison between results. Furthermore, no measures of occupational risk factors, and particularly psychosocial risk factors, that may impact on WA perception were included in the reviewed studies. It should be noted that self-reported WA can reflect, to a large extent, the person’s subjective view of their abilities (self-efficacy), thus being potentially affected by both physical and psychosocial occupational features.

Greater attention should be paid to exploring the overlap between individual factors and working conditions that may influence the perceived WA of employees. In this view, it could be interesting to achieve more information on sedentary job tasks offering PF and WA “background” data to be compared with those determined in more physically demanding jobs or in work with peculiar types of organization (ie, shift work). This may allow extrapolation of data primarily dependent on individual features avoiding possible influences derived from more complex occupational exposure settings. Moreover, it could be important to understand to what extent monitoring PF and WA perception can be useful to follow up the occupational health of potentially “susceptible” individuals, that is, due to aging or chronic diseases.

Overall, including PF tests in occupational health programs seems in line with the concept of sustainable employability and WA. It sounds coherent with the proposal of the US National Institute for Occupational Safety and Health (NIOSH), the NIOSH *Total Worker Health*, defined as policies, programs, and practices that integrate protection from work-related safety and health hazards with promotion of injury and illness-prevention efforts to advance worker well-being.^50^ To include PF and WA assessment in occupational health practice may represent a strategy to improve the well-being of workers by protecting their safety and enhancing their health and productivity.

In conclusion, evaluating the association between PF and WA is crucial for defining susceptible individuals, tailoring specific interventions to improve their PF, and preparing them to face their work-related tasks and occupational risks. This may be part of effective occupational health promotion strategies, aimed to promote the well-being of individuals at work and their sustainable WA. Some PF performance tests seem promising tools to include in workers’ health surveillance or promotion programs, although some issues need to be addressed first. It would be interesting to understand whether and to what extent an increase in PF may determine an improvement in WA. Furthermore, it remains difficult to use such PF tests in surveillance, as no definite cut-off values are available indicating low values related to poor WA. Research addressing these issues might be a useful next step to provide new insights into the relationship between workplace and individual related factors with respect to WA. This may be important to inform suitable strategies for occupational risk assessment and management, to promote health and well-being, and to favor an active aging of the workforce.

## Funding

Authors state no funding involved.

## Conflicts of interest

Authors state no conflict of interest.

## Data availability statement

Data sharing is not applicable to this article as no new data were created or analyzed in this study.

## Supplementary Material

Web_Material_uiad006
