# Supplementary material for: Association between health-related physical fitness indicators and working ability: a systematic review
Source: J Occup Health. 2023 Nov 8;66(1):uiad006. doi: 10.1093/joccuh/uiad006 (PMC11020302; doi:10.1093/joccuh/uiad006)
Supplement: Web_Material_uiad006 [file web_material_uiad006.zip › Supplementary material 1_Protocol and search strategy 2023_10_29.docx]

**Objectives of the review**

To explore the relationship between physical fitness (PF) measures and work ability (WA).

**Rationale of the review**

Work ability reflects a balance between work demands and an individual ability to meet them. It is influenced by several occupational and health related factors including the individual PF. If a worker's physical capacity is unable to meet the demands of the job, due to a progressive deterioration of various components of PF, he/she can suffer from a worse WA perception, thus leading to poor productivity and increased risk of workplace accidents. This seems even more important considering the aging of the workforce, a major concern for public health policies, which is responsible for health worsening, in terms of physiological and cognitive abilities, that may decrease WA.

**Protocol of the study**

The review protocol has not been registered in international databases.

**Changes to the protocol**

The initial, general search approach with the terms “physical fitness” and “work ability”, adopted to capture all the studies relevant for the scopes of the review was enlarged through the employment of search terms more specifically focusing on PF performance analysis as emerged from the preliminary search.

**Types of studies included**

The review includes cross-sectional and prospective cohort studies, published in English, addressing the influence of different PF parameters on the WA. A number of measurable components contribute to PF. These include balance, agility, coordination, speed, power, reactivity, as well as those components more strictly associated with health and well-being (health-related fitness), i.e. cardio-respiratory endurance, muscular endurance and strength, body composition, and flexibility. Therefore, we included studies investigating the possible impact that changes in measurable PF factors, e.g. aerobic capacity, walking speed, balance, flexibility, muscle strength and hand grip strength, may have on the WA of subjects employed in different occupational settings. Considering that the WA evaluation is a challenging issue and that many measures have been proposed, we have chosen to focus on studies using the Work Ability Index (WAI) or the Work Ability Score (WAS) questionnaires, known for their well-established predictive validity in WA assessment.

**Outcomes**

- To comprehensively assess the state of knowledge concerning the possible relationship between components of PF, including aerobic and muscular fitness, joint flexibility, as well as balance tasks, and WA.
- To extrapolate data eventually supporting the inclusion of PF assessment in occupational health practice as part of health and well-being promotion plans.
- To extrapolate useful information that may be useful to help workers to remain in the workforce and improve their productivity, supporting a long-term sustainable WA.

**Search strategy process**

Candidate search terms: “physical fitness” and “work ability” were identified looking at the words in the titles and abstracts of three known relevant studies focusing on the topic of the review. A draft search strategy was developed using those general terms in all the three explored databases. Such a general search approach was chosen to capture all the studies relevant for the scopes of the review. In all the three databases employed: Pubmed, Scopus, and ISI Web of Knowledge, the full line search strategy was: ("Physical fitness") AND ("work ability"). Nor limits or restrictions, nor search filters have been applied. Duplicate removal was manually performed reporting all the retrieved papers in an excel file. Additional search terms were identified from the results of the preliminary search strategy: ”aerobic capacity”, “walking speed”, “balance”, “flexibility”, “muscle strength” and “hand grip strength”. These were individually combined with the term “work ability”. The citation pool of relevant publications identified in the literature search was further enlarged by assessing the reference list accompanying the selected articles.

**Data extraction and analysis**

The full text of the eligible articles has been screened for inclusion by two researchers independently. In case of disagreement, in this phase, consensus on inclusion and exclusion was reached by discussion and, if necessary, a third researcher, was consulted. Key information about the included studies was collected in a standardized data extraction form independently by three of the authors and extracted data were then compared to exclude any possible inaccuracy during the process.

**Assessment of risk of bias in included studies**

Three of the Authors independently evaluated the quality of the selected studies using the Newcastle–Ottawa Quality Assessment Scale for case control and cohort studies or adapted for cross sectional ones. Based on a maximum of nine points attributable within three different sections, such as selection, comparability and outcome, a range scale was adopted, going from satisfactory studies with 5-6 points, good studies for 7–8 points and very good studies for 9-10 points. Scores lower than 4 categorized studies as unsatisfactory. When there was disagreement on the evaluation, the remaining authors also reviewed the article, and the judgement made by most of the reviewers determined the quality rating.

**Data synthesis**

The population of the reviewed studies was defined by reporting the number of workers considered in individual studies, age, sex, and type of work performed, including administrative and managerial work, “white-collar workers”, as well as "blue-collar" ones, including workers engaged in significant physical efforts at work. The anthropometric measurements and tests used to assess the PF performance and the score obtained were reported. Finally, the results of the individual studies were also summarized to provide the readers a suitable overview on the relationship between the investigated PF parameters and the perceived WA.

**Supplementary Table 1. Excluded studies and reason for exclusion.**

| **REFERENCES** | **REASON FOR EXCLUSION** |
| --- | --- |
| 1. Kettunen O, Vuorimaa T, Vasankari T. 12-mo intervention of physical exercise improved work ability, especially in subjects with low baseline work ability. Int J Environ Res Public Health. 2014 Apr 4;11(4):3859-3869. doi: 10.3390/ijerph110403859. | Out of topic for title and abstract analysis |
| 1. Vingård E, Blomkvist V, Rosenblad A, Lindberg P, Voss M, Alfredsson L, Josephson M. A physical fitness programme during paid working hours - impact on health and work ability among women working in the social service sector: a three year follow up study. Work. 2009;34(3):339-3244. doi: 10.3233/WOR-2009-0932. | Out of topic for title and abstract analysis |
| 1. Das Gecim GY, Esin MN. A self-management programme for work ability and quality of life in nurses aged 45 years and over: A randomized controlled trial. Int J Nurs Pract. 2021 Dec;27(6):e12963. doi: 10.1111/ijn.12963. | Out of topic for title and abstract analysis |
| 1. Bugajska J, Makowiec-Dabrowska T, Wagrowska-Koski E. Zarzadzanie wiekiem w przedsiebiorstwach jako element ochrony zdrowia starszych pracowników [Age management in enteprises as a part of occupational safety and health in elderly workers]. Med Pr. 2010;61(1):55-63. Polish. | Language other than English |
| 1. Goedhard WJ, Rijpstra TS, Puttiger PH. Age, absenteeism and physical fitness in relation to work ability. Stud Health Technol Inform. 1998;48:254-257. doi: 10.3233/978-1-60750-892-2-254. | Review articles or types of excluded papers |
| 1. Kenny GP, Groeller H, McGinn R, Flouris AD. Age, human performance, and physical employment standards. Appl Physiol Nutr Metab. 2016 Jun;41(6 Suppl 2):S92-S107. doi: 10.1139/apnm-2015-0483. | Review articles or types of excluded papers |
| 1. Chan G, Tan V, Koh D. Ageing and fitness to work. Occup Med (Lond). 2000 Sep;50(7):483-491. doi: 10.1093/occmed/50.7.483. | Out of topic for title and abstract analysis |
| 1. Ilmarinen J, Costa G. L'invecchiamento dei lavoratori nell'Unione Europea [Aging of the working population in the European Union]. Med Lav. 2000 Jul-Aug;91(4):279-95. Italian. PMID: 11098592. | Review articles or types of excluded papers |
| 1. Ilmarinen J, Costa G. L'invecchiamento dei lavoratori nell'Unione Europea [Aging of the working population in the European Union]. Med Lav. 2000 Jul-Aug;91(4):279-95. Italian. | Language other than English |
| 1. Tuomi K, Ilmarinen J, Martikainen R, Aalto L, Klockars M. Aging, work, life-style and work ability among Finnish municipal workers in 1981-1992. Scand J Work Environ Health. 1997;23 Suppl 1:58-65. | Out of topic for title and abstract analysis |
| 1. Przysada G, Kilian J, Wiśniowska-Szurlej A, Sozański B, Piwoński P, Drużbicki M, Wilmowska-Pietruszyńska A, Ćwirlej-Sozańska A. Ocena zdolności do podejmowania pracy po zakończeniu rehabilitacji w ramach prewencji rentowej Zakładu Ubezpieczeń Społecznych u osób z chorobami przewlekłymi narządu ruchu [An assessment of work ability after the completion of rehabilitation as part of disability pension prevention of the Social Insurance Institution among people with chronic musculoskeletal diseases]. Med Pr. 2019 Jul 16;70(4):459-473. Polish. doi: 10.13075/mp.5893.00826. | Language other than English |
| 1. Berglind D, Nyberg G, Willmer M, Persson M, Wells M, Forsell Y. An eHealth program versus a standard care supervised health program and associated health outcomes in individuals with mobility disability: study protocol for a randomized controlled trial. Trials. 2018 Apr 27;19(1):258. doi: 10.1186/s13063-018-2646-z. | Out of topic for title and abstract analysis |
| 1. Jebens E, Mamen A, Medbø JI, Knudsen O, Veiersted KB. Are elderly construction workers sufficiently fit for heavy manual labour? Ergonomics. 2015;58(3):450-462. doi: 10.1080/00140139.2014.977828. | Out of topic for title and abstract analysis |
| 1. Freude G, Seibt R, Pech E, Ullsperger P. Assessment of work ability and vitality-a study of teachers of different age groups. International Congress Series. 2005;1280: 270-274. Proceedings of 2nd International Symposium on Work Ability, 18-20 October 2004, Verona, Italy | Review articles or types of excluded papers |
| 1. Sörensen LE, Pekkonen MM, Männikkö KH, Louhevaara VA, Smolander J, Alén MJ. Associations between work ability, health-related quality of life, physical activity and fitness among middle-aged men. Appl Ergon. 2008 Nov;39(6):786-791. doi: 10.1016/j.apergo.2007.11.001. | Out of topic for title and abstract analysis |
| 1. Reiso H, Nygård JF, Jørgensen GS, Holanger R, Soldal D, Bruusgaard D. Back to work: predictors of return to work among patients with back disorders certified as sick: a two-year follow-up study. Spine (Phila Pa 1976). 2003 Jul 1;28(13):1468-1473. doi: 10.1097/01.BRS.0000067089.83472.1F. | Out of topic for title and abstract analysis |
| 1. Yoo J, Kim Y, Cho ER, Jee SH. Biological age as a useful index to predict seventeen-year survival and mortality in Koreans. BMC Geriatr. 2017 Jan 5;17(1):7. doi: 10.1186/s12877-016-0407-y. | Out of topic for title and abstract analysis |
| 1. Taulaniemi A, Kuusinen L, Tokola K, Kankaanpää M, Suni JH. Bio-psychosocial factors are associated with pain intensity, physical functioning, and ability to work in female healthcare personnel with recurrent low back pain. J Rehabil Med. 2017 Aug 31;49(8):667-676. doi: 10.2340/16501977-2261. | Out of topic for title and abstract analysis |
| 1. Santana JD, Mambrini JVD, Peixoto SV. Cardiorespiratory fitness and cardiometabolic risk factors among university professors. Rev Bras Med Esporte. 2018;24(2):102-106. doi: 10.1590/1517-869220182402171631 | Out of topic for title and abstract analysis |
| 1. Ilmarinen J, Tuomi K, Klockars M. Changes in the work ability of active employees over an 11-year period. Scand J Work Environ Health. 1997;23 Suppl 1:49-57. | Out of topic for title and abstract analysis |
| 1. Levushkin SP. Kompleksnaia otsenka fizicheskoĭ rabotosposobnosti iunosheĭ [Complex assessment of physical work ability of young men]. Fiziol Cheloveka. 2001 Sep-Oct;27(5):68-75. Russian. | Language other than English |
| 1. Gram B, Holtermann A, Bültmann U, Sjøgaard G, Søgaard K. Does an exercise intervention improving aerobic capacity among construction workers also improve musculoskeletal pain, work ability, productivity, perceived physical exertion, and sick leave?: a randomized controlled trial. J Occup Environ Med. 2012 Dec;54(12):1520-1526. doi: 10.1097/JOM.0b013e318266484a. | Out of topic for title and abstract analysis |
| 1. Witlox L, Schagen SB, de Ruiter MB, Geerlings MI, Peeters PHM, Koevoets EW, van der Wall E, Stuiver M, Sonke G, Velthuis MJ, Palen JAMV, Jobsen JJ, May AM, Monninkhof EM. Effect of physical exercise on cognitive function and brain measures after chemotherapy in patients with breast cancer (PAM study): protocol of a randomised controlled trial. BMJ Open. 2019 Jun 20;9(6):e028117. doi: 10.1136/bmjopen-2018-028117. | Out of topic for title and abstract analysis |
| 1. Nurminen E, Malmivaara A, Ilmarinen J, Ylöstalo P, Mutanen P, Ahonen G, Aro T. Effectiveness of a worksite exercise program with respect to perceived work ability and sick leaves among women with physical work. Scand J Work Environ Health. 2002 Apr;28(2):85-93. doi: 10.5271/sjweh.652. | Out of topic for title and abstract analysis |
| 1. Ohta M, Eguchi Y, Inoue T, Honda T, Morita Y, Konno Y, Yamato H, Kumashiro M. Effects of bench step exercise intervention on work ability in terms of cardiovascular risk factors and oxidative stress: a randomized controlled study. Int J Occup Saf Ergon. 2015;21(2):141-149. doi: 10.1080/10803548.2015.1029293. | Out of topic for title and abstract analysis |
| 1. Leino P, Kivekäs J, Hänninen K. Effects of work-oriented fitness courses in lumberjacks with low back pain. J Occup Rehabil. 1994 Jun;4(2):67-76. doi: 10.1007/BF02110046. | Out of topic for title and abstract analysis |
| 1. Pohjonen T, Ranta R. Effects of worksite physical exercise intervention on physical fitness, perceived health status, and work ability among home care workers: five-year follow-up. Prev Med. 2001 Jun;32(6):465-475. doi: 10.1006/pmed.2001.0837. | Out of topic for title and abstract analysis |
| 1. Lamminpää A, Kuoppala J, Väänänen-Tomppo I, Hinkka K. Employee and work-related predictors for entering rehabilitation: a cohort study of civil servants. J Rehabil Med. 2012 Jul;44(8):669-676. doi: 10.2340/16501977-1007. | Out of topic for title and abstract analysis |
| 1. Perkiö-Mäkelä M. Exercise and ergonomics-focused group counseling among female farmers. Occupational Ergonomics. 2001 Sep;2(4):239-250. doi: 10.3233/OER-2001-2404 | Out of topic for title and abstract analysis |
| 1. de Vries JD, van Hooff MLM, Geurts SAE, Kompier MAJ. Exercise to reduce work-related fatigue among employees: a randomized controlled trial. Scand J Work Environ Health. 2017 Jul 1;43(4):337-349. doi: 10.5271/sjweh.3634. Epub 2017 Mar 21. | Out of topic for title and abstract analysis |
| 1. Pugh JD, Cormack K, Gelder L, Williams AM, Twigg DE, Blazevich AJ. Exercise, fitness and musculoskeletal health of undergraduate nursing students: A cross-sectional study. J Adv Nurs. 2019 Oct;75(10):2110-2121. doi: 10.1111/jan.13990. | Out of topic for title and abstract analysis |
| 1. Perkio-Makela MM. Finnish farmers' self-reported morbidity, work ability, and functional capacity. Ann Agric Environ Med. 2000;7(1):11-16. | Out of topic for title and abstract analysis |
| 1. Tuomi K, Ilmarinen J, Klockars M, Nygård CH, Seitsamo J, Huuhtanen P, Martikainen R, Aalto L. Finnish research project on aging workers in 1981-1992. Scand J Work Environ Health. 1997;23 Suppl 1:7-11. | Out of topic for title and abstract analysis |
| 1. Ilmarinen J. Functional capacities and work ability as predictors of good 3rd age. In: Shiraki K, Sagawa S, Mohamed Yousef K, editors. Physical Fitness and Health Promotion in Active Ageing. Leiden, The Netherlands: Backhuys Publishers; 2001, p. 61-80. | Review articles or types of excluded papers |
| 1. Martinez MC, Latorre Mdo R. Saúde e capacidade para o trabalho de eletricitários do Estado de São Paulo [Health and work ability of workers of the electricity sector in São Paulo]. Cien Saude Colet. 2008 May-Jun;13(3):1061-1073. Portuguese. doi: 10.1590/s1413-81232008000300029. | Language other than English |
| 1. Langbrandtner J, Steimann G, Reichel C, Bokemeyer B, Hüppe A. Erwerbstätig mit chronisch entzündlicher Darmerkrankung –Herausforderungen im Arbeitsleben und unterstützende Maßnahmen zur Bewältigung [Inflammatory Bowel Disease - Challenges in the Workplace and Support for Coping with Disease]. Rehabilitation (Stuttg). 2022 Apr;61(2):97-106. German. doi: 10.1055/a-1581-6497. | Language other than English |
| 1. Perkiö-Mäkelä M. Influence of exercise-focused group activities on the physical activity, functional capacity, and work ability of female farmers--a three-year follow-Up. Int J Occup Saf Ergon. 1999;5(3):381-394. doi: 10.1080/10803548.1999.11076427. | Out of topic for title and abstract analysis |
| 1. Miettinen M, Louhevaara V. Job demands, physical fitness, work ability, and age of vehicle inspectors. 1994 Jun;13(4): 337-342. doi: 10.1016/0169-8141(94)90090-6. | Out of topic for title and abstract analysis |
| 1. Seitsamo J, Ilmarinen J. Life-style, aging and work ability among active Finnish workers in 1981-1992. Scand J Work Environ Health. 1997;23 Suppl 1:20-26. | Out of topic for title and abstract analysis |
| 1. Baranov VM, Solopov IN, Gorbaneva EP, Tamozhnikov DV, Iumatova SN, Kuznetsova TIu, Sentiabrev NN, Kamchatnikov AG, Tkachenko NV, Medvedev DV. [Optimization of directed training loads on the respiratory system]. Aviakosm Ekolog Med. 2008 Mar-Apr;42(2):36-9. Russian. | Language other than English |
| 1. Ojala B, Nygård CH, Nikkari ST. Outpatient rehabilitation as an intervention to improve employees' physical capacity. Work. 2016;55(4):861-871. doi: 10.3233/WOR-162458. | Out of topic for title and abstract analysis |
| 1. Bayrhuber M, Tinsel I, Goldacker S, Kindle G, Warnatz K, Farin E, Nieters A. Perceived health of patients with common variable immunodeficiency - a cluster analysis. Clin Exp Immunol. 2019 Apr;196(1):76-85. doi: 10.1111/cei.13252. | Out of topic for title and abstract analysis |
| 1. Sörensen L, Smolander J, Louhevaara V, Korhonen O, Oja P. Physical activity, fitness and body composition of Finnish police officers: a 15-year follow-up study. Occup Med (Lond). 2000 Jan;50(1):3-10. doi: 10.1093/occmed/50.1.3. | Out of topic for title and abstract analysis |
| 1. Prieske O, Dalager T, Looks V, Golle K, Granacher U. Physical fitness and psycho-cognitive performance in the young and middle-aged workforce with primarily physical versus mental work demands. J Public Health (Berl). 2021; 29: 75–84. doi:10.1007/s10389-019-01099-9 | Out of topic for title and abstract analysis |
| 1. Bugajska J, Sudoł-Szopińska I, Widerszal-Bazyl M. Physiological and psychosocial aspects of occupational activity of elderly workers. New Medicine. 2006 Oct;9(4):107-111. | Review articles or types of excluded papers |
| 1. Peršić V, Boban M, Laškarin G, Pehar-Pejčinović V, Miletić B, Brozina A, Travica D, Rakić M. Position of cardiovascular rehabilitation programs in the global burden of cardiovascular diseases. Medicina Fluminensis. 2012 Dec;48(4):395-402. | Review articles or types of excluded papers |
| 1. Astrand NE, Isacsson SO, Olhagen GO. Prediction of early retirement on the basis of a health examination. An 11-year follow-up of 264 male employees in a Swedish pulp and paper company. Scand J Work Environ Health. 1988 Apr;14(2):110-227. doi: 10.5271/sjweh.1948. | Out of topic for title and abstract analysis |
| 1. Gabrys L, Schmidt C. Verordnungshäufigkeit und Inanspruchnahme von sport- und bewegungstherapeutischen Leistungen in der kardiologischen Rehabilitation 2006–2013 [Prescription and Utilization of Sports Therapy Programs following Cardiac Rehabilitation 2006-2013]. Rehabilitation (Stuttg). 2020 Feb;59(1):42-47. German. doi: 10.1055/a-0869-9810. | Language other than English |
| 1. Perroni F, Guidetti L, Cignitti L, Baldari C. Psychophysiological responses of firefighters to emergencies: A review. Open Sports Sciences Journal. 2014; 7(SPEC.ISS1):8-15. doi: 10.2174/1875399x01407010008 | Review articles or types of excluded papers |
| 1. Baldwin JN, McKay MJ, Hiller CE, Moloney N, Nightingale EJ, Burns J. Relationship between physical performance and self-reported function in healthy individuals across the lifespan. Musculoskelet Sci Pract. 2017 Aug;30:10-17. doi: 10.1016/j.msksp.2017.05.001. | Out of topic for title and abstract analysis |
| 1. Plat MJ, Frings-Dresen MH, Sluiter JK. Reproducibility and validity of the stair-climb test for fire fighters. Int Arch Occup Environ Health. 2010 Oct;83(7):725-731. doi: 10.1007/s00420-010-0518-2. | Out of topic for title and abstract analysis |
| 1. Sun JJ. Research on Physical Education of Combining Health-related Physical Fitness and Specialties in Higher Vocational Colleges. Executive Chairman. 2018. | Review articles or types of excluded papers |
| 1. Leensen MCJ, Groeneveld IF, van der Heide I, Rejda T, van Veldhoven PLJ, Berkel SV, Snoek A, Harten WV, Frings-Dresen MHW, de Boer AGEM. Return to work of cancer patients after a multidisciplinary intervention including occupational counselling and physical exercise in cancer patients: a prospective study in the Netherlands. BMJ Open. 2017 Jun 15;7(6):e014746. doi: 10.1136/bmjopen-2016-014746. | Out of topic for title and abstract analysis |
| 1. Christensen JR, Kongstad MB, Sjøgaard G, Søgaard K. Sickness Presenteeism Among Health Care Workers and the Effect of BMI, Cardiorespiratory Fitness, and Muscle Strength. J Occup Environ Med. 2015 Dec;57(12):e146-152. doi: 10.1097/JOM.0000000000000576. | Out of topic for title and abstract analysis |
| 1. Costa G. Some considerations about aging, shift work and work ability. International Congress Series. 2005 Jun;1280:67-72. doi: 10.1016/j.ics.2005.02.088. Proceedings of 2nd International Symposium on Work Ability, 18-20 October 2004, Verona, Italy. | Review articles or types of excluded papers |
| 1. Mackey M, Maher CG, Wong T, Collins K. Study protocol: the effects of work-site exercise on the physical fitness and work-ability of older workers. BMC Musculoskelet Disord. 2007 Jan 31;8:9. doi: 10.1186/1471-2474-8-9. | Out of topic for title and abstract analysis |
| 1. Tuomi K, Ilmarinen J, Seitsamo J, Huuhtanen P, Martikainen R, Nygård CH, Klockars M. Summary of the Finnish research project (1981-1992) to promote the health and work ability of aging workers. Scand J Work Environ Health. 1997;23 Suppl 1:66-71. | Out of topic for title and abstract analysis |
| 1. Yang X, Telama R, Hirvensalo M, Hintsanen M, Hintsa T, Pulkki-Råback L, Mansikkaniemi K, Viikari JS, Keltikangas-Järvinen L, Raitakari OT. Sustained involvement in youth sports activities predicts reduced chronic job strain in early midlife. J Occup Environ Med. 2010 Dec;52(12):1154-1159. doi: 10.1097/JOM.0b013e3181fe68bf. | Out of topic for title and abstract analysis |
| 1. Ohta M, Okufuji T, Matsushima Y, Ikeda M. The effect of lifestyle modification on physical fitness and work ability in different workstyles. J UOEH. 2004 Dec 1;26(4):411-421. doi: 10.7888/juoeh.26.411. | Out of topic for title and abstract analysis |
| 1. Boocock MG, Mawston GA. The Effects of Repetitive Lifting on Heart Rate Responses and Perceived Exertion in a Young and Middle-aged Population. Advances in Physical Ergonomics and Safety. 2012; 12: 96-103. | Review articles or types of excluded papers |
| 1. van den Berg TI, Elders LA, de Zwart BC, Burdorf A. The effects of work-related and individual factors on the Work Ability Index: a systematic review. Occup Environ Med. 2009 Apr;66(4):211-220. doi: 10.1136/oem.2008.039883. | Review articles or types of excluded papers |
| 1. Brooke JD, Hamley EJ. The heart-rate--physical work curve analysis for the prediction of exhausting work ability. Med Sci Sports. 1972 Spring;4(1):23-26. | Out of topic for title and abstract analysis |
| 1. Schenk P, Klipstein A, Spillmann S, Strøyer J, Laubli T. The role of back muscle endurance, maximum force, balance and trunk rotation control regarding lifting capacity. Eur J Appl Physiol. 2006 Jan;96(2):146-156. doi: 10.1007/s00421-004-1262-7. | Out of topic for title and abstract analysis |
| 1. Strijk JE, Proper KI, van Stralen MM, Wijngaard P, van Mechelen W, van der Beek AJ. The role of work ability in the relationship between aerobic capacity and sick leave: a mediation analysis. Occup Environ Med. 2011 Oct;68(10):753-758. doi: 10.1136/oem.2010.057646. | Out of topic for title and abstract analysis |
| 1. Mackey MG, Bohle P, Taylor P, Di Biase T, McLoughlin C, Purnell K. Walking to wellness in an ageing sedentary university community: design, method and protocol. Contemp Clin Trials. 2011 Mar;32(2):273-279. doi: 10.1016/j.cct.2010.12.001. | Out of topic for title and abstract analysis |
| 1. El Fassi M, Bocquet V, Majery N, Lair ML, Couffignal S, Mairiaux P. Work ability assessment in a worker population: comparison and determinants of Work Ability Index and Work Ability score. BMC Public Health. 2013 Apr 8;13:305. doi: 10.1186/1471-2458-13-305. | Out of topic for title and abstract analysis |
| 1. Wendel H. Arbeitsfähigkeit bei Sarkoidose [Work ability in sarcoidosis]. Z Gesamte Inn Med. 1971 Sep 15;26(18):252-6. German. | Language other than English |
| 1. Juszczyk G, Czerw AI, Religioni U, Olejniczak D, Walusiak-Skorupa J, Banas T, Mikos M, Staniszewska A. Work Ability Index (WAI) values in a sample of the working population in Poland. Ann Agric Environ Med. 2019 Mar 22;26(1):78-84. doi: 10.26444/aaem/91471. | Out of topic for title and abstract analysis |
| 1. Gregio Neto N, Spröesser Alonso M, Bernardes JM, Ruiz-Frutos C, Gómez-Salgado J, Dias A. Work ability of informal caregivers of patients treated by the public home care service of Brazil: A cross-sectional study. Safety Science. 2021 Dec; 144: Article number 105472. doi: 10.1016/j.ssci.2021.105472. | Out of topic for title and abstract analysis |
| 1. Ünlü H, Filiz B. Work Ability of the Turkish Physical Education Teachers. Res Q Exerc Sport. 2019 Dec;90(4):666-677. doi: 10.1080/02701367.2019.1642995. | Out of topic for title and abstract analysis |
| 1. Laitinen J, Ek E, Tammelin T, Pekkarinen A, Mielonen P, Anttonen H, Rintamäki H. Work ability of young adults. Int J Circumpolar Health. 1998 Jul;57(2-3):162-169. | Review articles or types of excluded papers |
| 1. Smolander J, Blair SN, Kohl HW 3rd. Work ability, physical activity, and cardiorespiratory fitness: 2-year results from Project Active. J Occup Environ Med. 2000 Sep;42(9):906-910. doi: 10.1097/00043764-200009000-00012. | Out of topic for title and abstract analysis |

**Supplementary Table 1. Included studies from the preliminary online search**

| 1. Sörensen L, Honkalehto S, Kallinen M, Pekkonen M, Louhevaara V, Smolander J, Alén M. Are cardiorespiratory fitness and walking performance associated with self-reported quality of life and work ability? Int J Occup Med Environ Health. 2007;20(3):257-264. doi: 10.2478/v10001-007-0023-3. |
| --- |
| 1. Pohjonen T. Age-related physical fitness and the predictive values of fitness tests for work ability in home care work. J Occup Environ Med. 2001 Aug;43(8):723-730. doi: 10.1097/00043764-200108000-00011. |
| 1. Smolander J, Sörensen L, Pekkonen M, Alén M. Muscle performance, work ability and physical functioning in middle-aged men. Occup Med (Lond). 2010 Jan;60(1):78-80. doi: 10.1093/occmed/kqp122. |
| 1. Ezzatvar Y, Calatayud J, Andersen LL, Vieira ER, López-Bueno R, Casaña J. Muscular Fitness and Work Ability among Physical Therapists. Int J Environ Res Public Health. 2021 Feb 10;18(4):1722. doi: 10.3390/ijerph18041722. |
| 1. Lebde N, Burns J, Mackey M, Baldwin J, McKay M. Normative reference values and physical factors associated with work ability: a cross-sectional observational study. Occup Environ Med. 2020 Apr;77(4):231-237. doi: 10.1136/oemed-2019-106248. |
| 1. Kaleta D, Makowiec-Dabrowska T, Jegier A. Leisure-time physical activity, cardiorespiratory fitness and work ability: a study in randomly selected residents of Lódź. Int J Occup Med Environ Health. 2004;17(4):457-464. |
| 1. Berner C, Haider S, Grabovac I, Lamprecht T, Fenzl KH, Erlacher L, Quittan M, Dorner TE. Work Ability and Employment in Rheumatoid Arthritis: A Cross-Sectional Study on the Role of Muscle Strength and Lower Extremity Function. Int J Rheumatol. 2018 Aug 1;2018:3756207. doi: 10.1155/2018/3756207. |
